# A founder *UMOD* variant is a common cause of hereditary nephropathy in the British population

**DOI:** 10.1136/jmg-2022-108704

**Published:** 2022-08-29

**Authors:** Manoj K Valluru, Noelle KX Chung, Mark Gilchrist, Laura Butland, Jackie Cook, Anna Takou, Abhijit Dixit, Michael N Weedon, Albert C M Ong, The Genomics England Research Consortium, John C Ambrose

**Affiliations:** 1 Academic Nephrology Unit, Department of Infection, Immunity and Cardiovascular Disease, The University of Sheffield Medical School, Sheffield, UK; 2 College of Medicine and Health, University of Exeter, Exeter, UK; 3 Department of Clinical Genetics, Nottingham University Hospitals NHS Trust, Nottingham, UK; 4 Department of Clinical Genetics, Sheffield Children's Hospital NHS Foundation Trust, Sheffield, UK; 5 Department of Histopathology, Sheffield Teaching Hospitals NHS Foundation Trust, Sheffield, UK; 6 Institute of Biomedical and Clinical Science, University of Exeter, Exeter, UK; 7 Sheffield Kidney Institute, Sheffield Teaching Hospitals NHS Foundation Trust, Sheffield, UK

**Keywords:** founder effect, genetics, population, mutation, genetic counseling, diagnosis

## Abstract

**Background:**

Monogenic disorders are estimated to account for 10%–12% of patients with kidney failure. We report the unexpected finding of an unusual uromodulin *(UMOD)* variant in multiple pedigrees within the British population and demonstrate a shared haplotype indicative of an ancestral variant.

**Methods:**

Probands from 12 apparently unrelated pedigrees with a family history of kidney failure within a geographically contiguous UK region were shown to be heterozygous for a pathogenic variant of *UMOD* c.278_289delTCTGCCCCGAAG insCCGCCTCCT.

**Results:**

A total of 88 clinically affected individuals were identified, all born in the UK and of white British ethnicity. 20 other individuals with the variant were identified in the UK 100,000 Genomes (100K) Project and 9 from UK Biobank (UKBB). A common extended haplotype was present in 5 of the UKBB individuals who underwent genome sequencing which was only present in <1 in 5000 of UKBB controls. Significantly, rare variants (<1 in 250 general population) identified within 1 Mb of the *UMOD* variant by genome sequencing were detected in all of the 100K individuals, indicative of an extended shared haplotype.

**Conclusion:**

Our data confirm a likely founder *UMOD* variant with a wide geographical distribution within the UK. It should be suspected in cases of unexplained familial nephropathy presenting in patients of white British ancestry.

**WHAT IS ALREADY KNOWN ON THIS TOPIC**

- A rare uromodulin *(UMOD)* variant was first reported in a UK pedigree with familial nephropathy in 2001, followed by isolated reports.
- A previous study concluded that the variant was a recurrent change rather than a founder effect.

**WHAT THIS STUDY ADDS**

- A further 41 pedigrees with the rare *UMOD* variant identified through regional centres and large national databases.
- Compelling evidence for a pathogenic ancestral variant highly prevalent in the white British population, responsible for kidney failure and chronic kidney disease.

**HOW THIS STUDY MIGHT AFFECT RESEARCH, PRACTICE OR POLICY**

- A high index of clinical suspicion in undiagnosed cases of familial nephropathy of white British ancestry.
- Definition of a large affected group of common ancestry sharing the same variant and thus opportunities to study non-allelic contributions to phenotypic variability.

## Introduction

Chronic kidney disease (CKD) is estimated to affect up to 10% of the world’s population, making it a leading cause of morbidity and mortality.^1^ Although CKD and kidney failure usually results from the interaction of lifestyle or environmental factors with complex polygenic traits, monogenic disorders are estimated to account for 10%–12% of kidney failure.^2^ The vast majority of these cases can be attributed to autosomal dominant polycystic kidney disease but pathogenic gene variants especially associated with autosomal dominant tubulointerstitial kidney disease (ADTKD) and Alport syndrome are being increasingly recognised.^2^

ADTKD is a rare genetic cause of progressive CKD and kidney failure. It is genetically heterogenous with pathogenic variants in *MUC1*,^3^ *REN*,^4^ *HNF1B* 5 and uromodulin *(UMOD)* 6 identified, with *UMOD* comprising the highest proportion of ADTKD cases of up to 50%, with a disease prevalence of 9 per million.^7^ ADTKD-*UMOD* is estimated to account for 2% of patients with kidney failure,^8^ with susceptibility *UMOD* variants also conferring around 20% increased risk for CKD and 15% for hypertension.^9^ Classically, it is characterised by early onset gout, hyperuricaemia, the absence of haematuria or proteinuria and kidney failure usually occurring between 30 and 60 years.

*UMOD* encodes uromodulin, the most abundant mammalian urinary protein, which is predominantly produced by the thick ascending limb of the loop of Henle in the renal tubule.^10^ Uromodulin contains an N-terminal signal peptide, three epidermal growth factor-like (EGF-like) domains, an eight-cysteine domain (D8C), a zona pellucida (ZP) domain and a glycosylphosphatidylinositol anchor segment. Although the physiological roles of uromodulin have not been fully established, studies on *UMOD* knockout mice have suggested protective roles against urinary tract infections (UTIs),^11–13^ calcium oxalate formation^14^ and in facilitating electrolyte reabsorption in the renal tubules.^15 16^ Pathogenic *UMOD* variants lead to protein misfolding resulting in intracellular accumulation within the endoplasmic reticulum (ER)^17^ and a subsequent reduction in urinary secretion.^18^ Over 100 distinct *UMOD* variants have been reported to contribute to ADTKD-*UMOD*, with 95% localising in exon 3 and exon 4.^19^ The clinical presentation across ADTKD-*UMOD* genotypes appears to be variable with no clear genotype-phenotype correlation.^20^

In this study, we report a rare *UMOD* variant, c.278_289delTCTGCCCCGAAG insCCGCCTCCT detected in a cluster of unrelated families within a contiguous geographical area with unexplained CKD leading to kidney failure. In national UK cohorts (100,000 Genomes (100K) Project and UK Biobank (UKBB)), we detected other cases with the same variant from other geographical regions but who share a common extended haplotype indicative of an ancestral mutation originating in the UK population.

## Materials and methods

### Study population

All probands were referred to the renal genetic clinics at Sheffield and Nottingham due to a family history of kidney failure of unknown aetiology. Index cases with heterozygous *UMOD* variant c.278_289delTCTGCCCCGAAG insCCGCCTCCT had their family history assessed. Affected relative with CKD and at-risk, apparently healthy family members were subsequently invited to a renal genetics consultation where they were offered genetic testing and had their clinical features, kidney function and kidney morphology (ultrasound) assessed. The age of onset of kidney failure was recorded and patients without kidney failure had their latest estimated glomerular filtration rate (eGFR) estimated using the Chronic Kidney Disease Epidemiology Collaboration (CKD-EPI) 2009 equation. The definition and staging of CKD followed the Kidney Disease Outcomes Quality Initiative and Kidney Disease Improving Global Outcomes (KDIGO) guidelines. CKD was defined as the presence of an eGFR <60 mL/min/1.73 m^2^ or the presence of markers indicating kidney damage, such as albuminuria for >3 months. Staging of CKD was based on eGFR and severity of albuminuria, and their CKD class specified according to KDIGO guidelines.^21 22^ A retrospective review of all patients screened for the *UMOD* variant at both renal genetic clinics was conducted.

### Mutational analysis

The recurrent pathogenic *UMOD* variant c.278_289delTCTGCCCCGAAG insCCGCCTCCT (exon 3) was identified using targeted analysis or panel-based sequencing in familial cases of kidney failure. Five of the index cases were identified on a six gene ADTKD next-generation sequencing gene panel offered through the Sheffield Diagnostic Genetics Service comprising the following genes: *REN, UMOD, HNF1B, SEC61A1, TSC1, TSC2* and another five through a two gene panel (*UMOD, REN*) offered through Oxford University Hospitals. The remaining two cases were diagnosed on direct *UMOD* testing or on a 15 gene tubulointerstitial kidney disease panel (R202, Panel App V.1.3) which includes *UMOD* (Bristol Genetics Laboratory).

### Genomics England 100,000 Genomes Project

Inclusion and genotyping of participants in the 100K was managed by Genomics England Limited (GEL). All participants in 100K provided written consent to access their anonymised clinical and genomic data for research purposes (https://re.extge.co.uk/ovd/). Whole-genome sequencing (WGS) was performed on all participants and processed using the GEL rare disease analysis pipeline as previously described.^23 24^ Phenotypes of identified carriers were manually reviewed in the Genomics England Participant Explorer. Initially, affected candidates were filtered based on c.278_289delTCTGCCCCGAAG insCCGCCTCCT *UMOD* variant (rs878855325) in the interactive variant analysis browser (IVA V.2.0, CG38 and RD38). Further detailed analysis of rs878855325 for the selected cases was extracted from the IVA browser and phenotype information from the KIBANA Data discovery (V.3.2) browser. KIBANA and IVA are part of the secured access environment. Cohort statistics were expressed as very rare (frequency <0.1%), rare (frequency <0.5%), average (frequency <5%), common (frequency >5%) or not observed.^25^ Conservation was assessed as follows: PhyloP score (positive scores—measure conservation, which is slower evolution than expected, at sites that are predicted to be conserved), PhastCons (scores represent probabilities of negative selection and range between 0 and 1) and Genomic Evolutionary Rate Profiling (score ranges from −12.3 to 6.17, with 6.17 being the most conserved). Selected cases and phenotype history were recorded in an excel file using a pseudo-case ID. Finally, recorded data were subjected to downstream bioinformatics analysis.

### UK Biobank

The UKBB comprises approximately 500 000 participants with extensive phenotyping and genetic data linked to clinical care records.^26^ The whole-exome sequencing pipeline and quality control has been recently described.^27^ We examined the available UKBB cohort exome data for the presence of the c.278_289delTCTGCCCCGAAG insCCGCCTCCT variant in the *UMOD* gene using the NM_003361.3 transcript and obtained demographic data from baseline assessment including age and sex. Clinical phenotype data relevant to ADTKD-*UMOD* including CKD-EPI eGFR, systolic and diastolic blood pressure, albumin:creatinine ratio and serum urate concentration from enrolment in UKBB were obtained along with Hospital Episode Statistics (HES) data for CKD and gout. Ancestry was determined by self-reporting at the assessment centre. Statistical differences in clinical data were determined by independent t-test for continuous data and a Fisher’s exact test for categorical data.

### Haplotype analysis

We used the directly genotyped SNP chip data from UKBB to phase haplotypes on chromosome 16. The genotyping and quality control of UKBB has been described previously.^26^ We only used SNPs with a minor allele frequency >5% with a missingness rate <1%. We used SHAPEIT2^28^ for haplotype phasing.

To support the common variant haplotype analyses, we used WGS data available in 150 000 UKBB individuals. Five of the nine individuals with the c.278_289delTCTGCCCCGAAG insCCGCCTCCT variant had WGS data available. The genome sequencing and quality control have been described in detail elsewhere (doi: https://doi.org/10.1101/2021.11.16.468246).

We identified four rare variants (<0.5% frequency in the UKBB) in the 1 Mb window around c.278_289delTCTGCCCCGAAG insCCGCCTCCT and tested whether they were associated with the presence of the *UMOD* variant using Fisher’s exact test. We determined ancestry using principal component analysis as previously described.^29^

We then assessed the frequency of four rare variants associated with c.278_289delTCTGCCCCGAAG insCCGCCTCCT in the UKBB in the 100K participants. The genome sequencing and quality control have been described previously.^24^ We used Fisher’s exact test to test for an increased frequency of these variants in the c.278_289delTCTGCCCCGAAG insCCGCCTCCT heterozygotes compared with the 100K background population.

### In silico studies

The three-dimensional (3D) structure of uromodulin (UniProt: P07911) was downloaded from AlphaFold DB (https://alphafold.ebi.ac.uk/). An experimentally validated indel mutant structure is not available, and therefore we generated a mutant structure by introducing the indel mutation in silico, computationally modelled by AlphaFold Colab (https://github.com/deepmind/alphafold).^30^ All types of direct interactions: polar and non-polar, favourable and unfavourable, including clashes, were analysed using contacts command in UCSF Chimera V.1.14.^31^ In the output, the atom-atom contacts are listed in order of decreasing van der Waals (VDW) overlap: positive where the atomic VDW spheres are intersecting, zero if just touching and negative if separated by space. The superimposed 3D structural model of the EGF II domain was obtained by superimposing mutated EGF II into the EGF II of the UMOD model, using the MatchMaker tool on UCSF Chimera V.1.14.^31^ The evolutionary conservation score of each amino acid of UMOD (EGF II domain) was determined using the ConSurf algorithm, based on the phylogenetic relationships between sequence homologues.^32^ The predicted impact of the mutation on protein function was analysed using DeepFRI (Graph Convolutional Network for predicting protein functions).^33^

### Maps and plots

The bubble and choropleth map plots were created in R studio (R V.4.0.5). Shapefile: NUTS Level 2 (Counties) Boundaries file was downloaded from geoportal.statistics.gov.uk (valid as of January 2018). A Sankey diagram was created using packages Canvg and d3.js (https://github.com/nowthis/sankeymatic). Pedigree structures were plotted manually in Microsoft PowerPoint.

### Statistical analysis

Descriptive measures were presented as mean±SD or median (IQR or range as specified). Renal survival was defined as time to the start of renal replacement therapy and was displayed as Kaplan-Meier survival plots. Patients were censored if they did not receive renal replacement therapy in the study duration and a log-rank test was used for comparison between sex. A χ^2^ test was used to determine association between hypertensive status and the development of kidney failure. A p value of <0.05 was deemed as significant. All statistical analysis was done with SPSS V.26.

## Results

### Clinical findings at presentation

#### Genetic testing

A total of 91 patients across 12 families with available clinical data were reviewed and their diagnostic outcomes were summarised (online supplemental figure 1). Altogether, there were 88 patients with definite or likely ADTKD-*UMOD*, 38 with a genetic diagnosis and 50 with a presumptive diagnosis (not genotyped) based on their clinical characteristics and family history according to diagnostic criteria laid out by the KDIGO Consensus Report on ADTKD.^34^ Of the latter, 94% (47/50) had a recorded diagnosis of kidney failure and 66% (33/50) were deceased.

10.1136/jmg-2022-108704.supp1 Supplementary data

#### Kidney function

Online supplemental table 1 shows the breakdown of clinical features in the patients with ADTKD-*UMOD*. Eighty-two per cent (71/88) had a diagnosis of CKD with 69% (61/88) having kidney failure. The median age of kidney failure was 52 years (range 32–76). The remaining 31% (27/88) who did not have CKD on presentation were younger and had a median age of 32.5 years (range 17–73). The median age of kidney survival was 55 years with no significant sex difference (p=0.31 by log-rank test) based on Kaplan-Meier survival analysis (online supplemental figure 2). Within each family however, the age of kidney failure varied greatly between individuals with a median age of 52 (range 32–76) years; pedigree 12 had no information on individuals reaching kidney failure (online supplemental figure 3).

#### Clinical features

Hypertension was present in 65% (30/46) of our cohort with available blood pressure readings. Hypertension showed a non-significant association with the development of kidney failure (p=0.07). Proteinuria was rarely seen and only three patients had proteinuria ≥30 mg/g. Microscopic haematuria (trace or 1+) was detected in 18% (7/40); no case of macroscopic haematuria was reported. Where available, hyperuricaemia was noted in 32% (9/28) at presentation and 61% (17/28) had transient hyperuricaemia recorded on at least one occasion. Nonetheless, gout was not a prominent feature, with only 6.9% (6/87) reporting incident gout at a median age onset of 42.5 (range 29–61) years. Only two patients had renal stones, one was asymptomatic and diagnosed incidentally. Of patients with available information, 15% (8/55) reported an episode of UTI during their lifetime; recurrent UTIs were present in 4% (2/55).

#### Renal ultrasound

Among patients with available renal ultrasound results (n=28), 61% (17/28) had normal-sized kidneys and the remaining 39% (11/28) had small kidneys. Renal cysts were uncommon: 18% (5/28) had either one or two cysts in each kidney and only one patient had >10 cysts in each kidney.

#### Biopsy findings

Eight patients with the variant had renal biopsies and only one patient had a normal biopsy report (online supplemental table 1). Among biopsies with abnormal findings, interstitial fibrosis and tubular atrophy were the most common findings seen: examples from two patients are shown in online supplemental figure 4. Arterial profiles were mostly thick walled, hyalinosis was rare and seen in only one patient. There was an absence of immune reactants on immunofluorescence. All glomerular basement membranes visualised on electron microscopy were reported to be normal. In one patient (3.5), an enlargement in lysosomes was seen in podocytes.

#### Overview of the clinical pedigrees presenting to renal genetic clinics

A total of 53 individuals from Sheffield (6 pedigrees) and 35 individuals from Nottingham (6 pedigrees) were identified. The index cases from each of the 12 pedigrees presented at a median age of 50 (range 26–60 years), following a referral from either the renal team or general practice. On initial presentation, all had an eGFR <60 mL/min/1.73 m^2^, with the majority having a known diagnosis of hypertension (n=9). Across the Sheffield and Nottingham cohorts, a family history of CKD or kidney failure was present in up to three generations in one pedigree, four generations in nine pedigrees and five generations in two pedigrees. Details of all 12 pedigrees (SN1–12) are summarised in online supplemental figures 5 and 6.

### The c.278_289delTCTGCCCCGAAG insCCGCCTCCT variant in *UMOD* leads to a predicted change in EGF II domain structure and altered Ca^2+^ binding

**Figure 1:**
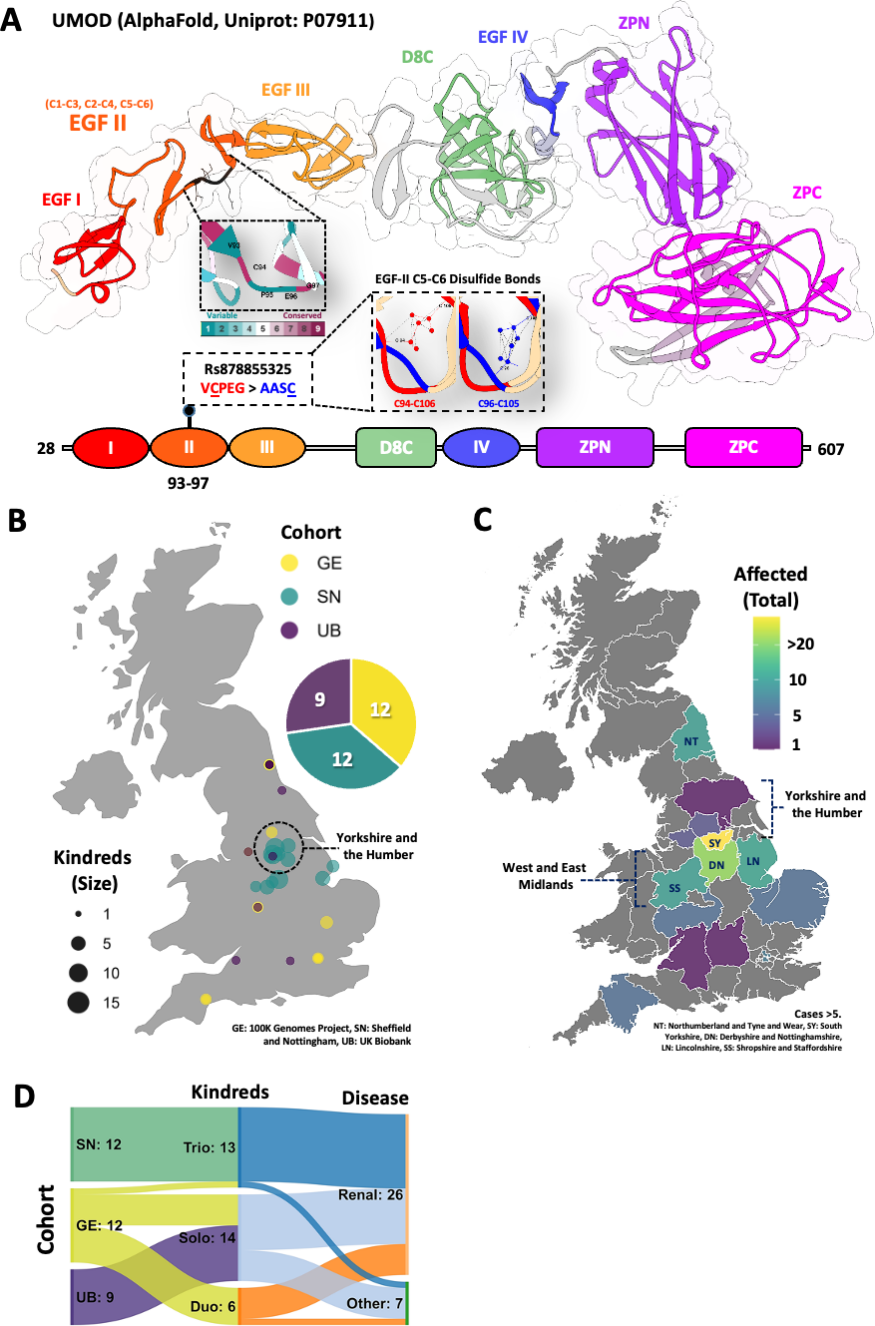
Structural model of wild-type and mutant uromodulin (UMOD) and demographics of the c.278_289delTCTGCCCCGAAG insCCGCCTCCT *UMOD* variant (Indel rs878855325). (A) AlphaFold structure and schematic diagram of uromodulin contains four EGF-like (I–IV), D8C, ZPN and ZPC domains. Indel rs878855325 (VCPEG>AASC) in the EGF II domain (highly conserved C94 and G97 residues) can affect structural integrity by disrupting disulfide bonds in C5-C6 (red: wild type and blue: mutant). (B) UK bubble map showing cohorts of unique pedigrees with Indel rs878855325. (C) UK choropleth map showing total identified cases across different counties in England (cases >5; NT, Northumberland and Tyne and Wear; SY, South Yorkshire; DN, Derbyshire and Nottinghamshire; LN, Lincolnshire; SS, Shropshire and Staffordshire). (D) Sankey diagram of Indel rs878855325 cohorts with paired cohort group and kindreds (trio, duo and solo or singleton). Disease status is indicated (renal and other). GE, 100K Genomes Project, SN, Sheffield and Nottingham; UB, UK Biobank.

The mature UMOD protein is 616 amino acids in length. The predicted structure of UMOD contains four EGF-like domains (EGF I–IV), a cysteine-rich D8C domain and a bipartite C-terminal ZP domain (ZPN and ZPC) (figure 1A). Domains EGF II and EGF III are predicted to bind Ca^2+^.

The specific indel variant results in the replacement of five amino acids ‘VCPEG’, by four novel residues ‘AASC’. DeepFRI results were: EGF II wild type—GO:0043169 score=0.84 and GO:0005509 score=0.54 and indel—GO:0043169 score=0.60. Based on in silico predictions using AlphaFold and DeepFRI, this change is predicted to alter protein folding and structural integrity of the EGF II domain and could alter the dynamics of Ca^2+^ binding and protein polymerisation.

### The c.278_289delTCTGCCCCGAAG insCCGCCTCCT variant is present in 20 individuals with and without a renal phenotype in the 100,000 Genome Project (GEL)

To identify potential cases from a wider geographical area in England, we accessed the 100K database and identified the c.278_289delTCTGCCCCGAAG insCCGCCTCCT variant (Indel rs878855325, ClinVar 242346) in 20 individuals (online supplemental table 3). Within the RD38 (rare disease) cohort, 17 individuals were identified across 9 families (GE1–9), with 6 families recruited for kidney phenotypes and 3 for non-kidney phenotypes (online supplemental figure 7). A further three individuals were identified within the CG38 (cancer germline) cohort but without additional phenotypic information. The calculated allele frequency was 0.0001582 (0.01582%) for the RD38 population and 0.00009841 (0.009841%) for the CG38 population. We did not identify any positive cases in The Cancer Genome Atlas (PanCancer Atlas Studies, cBioPortal V.4.1.9) and The Genome Aggregation Database (GnomAD V.3.1.2).

### Nine individuals are heterozygous for the c.278_289delTCTGCCCCGAAG insCCGCCTCCT variant in UK Biobank

**Figure 2:**
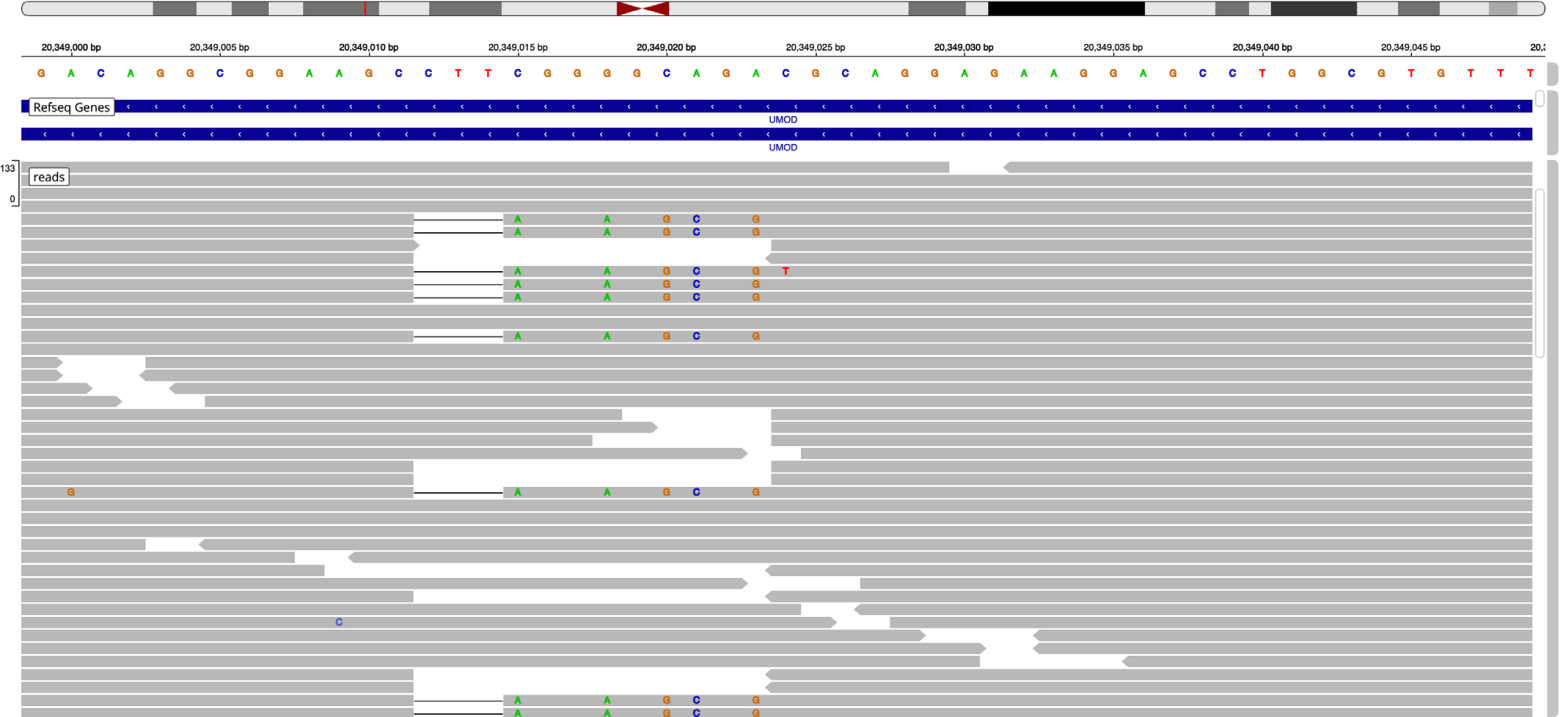
IGV plot showing the c.278_289delTCTGCCCCGAAG insCCGCCTCCT uromodulin *(UMOD)* variant. It has been miscalled as six separate variants.

Out of 450 993 individuals in the UKBB with exome sequencing data, 9 individuals, 7 males, 2 females were found to possess a single copy of the c.278_289delTCTGCCCCGAAG insCCGGCTCCT variant (table 1). This variant had been miscalled as six separate variants, but visualisation of Integrative Genomics Viewer (IGV) plots of individuals with these variants showed that individuals with these six variants all represent the single p.Val93_Gly97delinsAlaAlaSerCys variant (figure 2). Although we were unable to confirm these cases by Sanger sequencing, we noted that the same pattern was observed in the 100K individuals with this variant, and all have been confirmed by Sanger sequencing. No other *UMOD* variants were identified in these individuals.

**Table 1:** Clinical and laboratory features of individuals with the *UMOD* indel variant compared with others in the UKBB cohort

	*UMOD* indel (n=9)	UKBB (n=450 993)	P value
Age (years)	58.5±8.7	57.3±8.0	0.69
Sex (M/F %)	77.8/22.2	54.3/45.7	0.194
eGFR CKD-EPI (mL/min/1.73 m^2^)	69.0±20.3	90.5±13.0	**0.016**
ACR (mg/mmol)	3.1±5.0	1.6±2.5	0.42
SBP (mm Hg)	163.6±20.8	144.2±24.1	**0.023**
DBP (mm Hg)	99.3±13.0	86.4±13.5	**0.017**
Uric acid (μmol/L)	309.7±55.1	309.1±80.4	0.98
Gout	0	9338	1.0

Bold values are statistically significant ie p < 0.05.

ACR, albumin:creatinine ratio; CKD-EPI, Chronic Kidney Disease Epidemiology Collaboration; DBP, diastolic blood pressure; eGFR, estimated glomerular filtration rate; F, female; M, male; SBP, systolic blood pressure; UKBB, UK Biobank; UMOD, uromodulin.

All nine individuals were of European descent. Seven were unrelated and two were known to be related to each other. Kidney function was lower in those with the variant: CKD-EPI eGFR 69.0±20.3 vs 90.5±13.0 mL/min/1.73 m² (p=0.016). Systolic blood pressure was higher: 163.6±20.8 vs 144.2±24.1 mm Hg (p=0.023), as was diastolic blood pressure: 99.3±13.0 vs 86.4±13.5 mm Hg (p=0.017). In keeping with previous reports, gout was absent in those with this variant. Serum urate concentration did not differ between those with the variant and the background population 309.7±55.1 vs 309.1±80.4 µmol/L (p=0.98). Only one individual had no evidence of CKD or hypertension (age 43.3 years at baseline). Of the remaining eight (all aged >48 years at baseline), all had hypertension with seven having evidence of CKD (based on HES codes or CKD-EPI eGFR <60 mL/min/1.73 m² at baseline assessment). The age of those carrying the variant did not differ from the background population: 58.5±8.7 vs 57.3±8.0, p=0.69.

### Geographical distribution

In total, 117 individuals with the indel variant were identified from the 3 cohorts. Of interest, individual cases were found to originate from several geographical regions across England (figure 1B). Five regions had more than five cases with the highest concentration of cases in South Yorkshire (figure 1C). For the 100K and UKBB cohorts however, wider family history data were not available leading to a potential underestimation of affected individuals in these pedigrees (figure 1D).

### Individuals with c.278_289delTCTGCCCCGAAG insCCGCCTCCT share an extended haplotype

**Figure 3:**
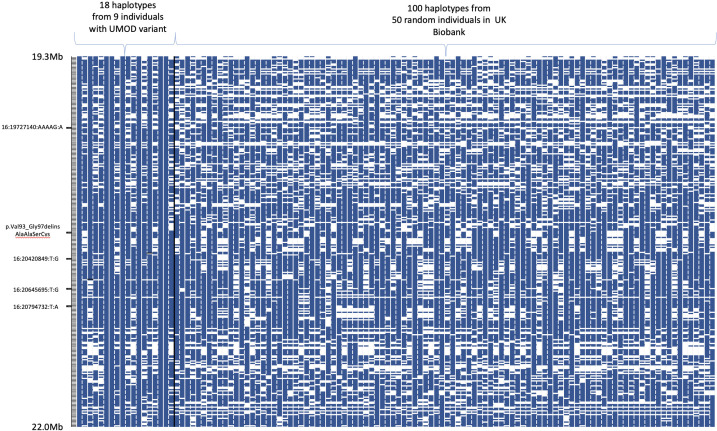
Haplotype analysis of phased common variants demonstrates all nine individuals in the UK Biobank with c.278_289delTCTGCCCCGAAG insCCGCCTCCT carry a shared extended haplotype. Blue indicates where an allele matches the putative shared haplotype for that variant and white where it does not match. The four rare variants identified from whole-genome sequencing are indicated on the left-hand side of the figure. UMOD, uromodulin.

All five cases in UKBB with WGS were from the group comprising white British or Irish ancestry (88%) and all nine individuals in UKBB were of European ancestry by principal component analysis. Only two of the individuals were >third-degree relatives. The nine individuals shared a haplotype around c.278_289delTCTGCCCCGAAG insCCGCCTCCT based on phasing directly genotyped common SNPs from the UKBB SNP chip (figure 3). The shared haplotype extends from 7 Mb to >30 Mb between individuals. This minimal shared haplotype occurs in <1 in 5000 of the remaining UKBB samples.

Five of the individuals in UKBB had undergone WGS. In the 1 Mb window around the c.278_289delTCTGCCCCGAAG insCCGCCTCCT variant, there were four rare variants present in individuals with the c.278_289delTCTGCCCCGAAG insCCGCCTCCT, which were rarely found in the general population (present in <1 in 250 individuals; table 2): all individuals with c.278_289delTCTGCCCCGAAG insCCGCCTCCT carried at least one of these variants. We then assessed whether the 20 individuals confirmed to carry c.278_289delTCTGCCCCGAAG insCCGCCTCCT in the 100K also carried these rare variants. All carried at least one of these rare variants, and most all four (table 2). This demonstrates a shared haplotype across all individuals with c.278_289delTCTGCCCCGAAG insCCGCCTCCT.

**Table 2:** Rare variant analysis in UKBB and 100K (GEL) whole-genome sequencing datasets provides strong evidence for shared haplotype in all individuals with the c.278_289delTCTGCCCCGAAG insCCGCCTCCT variant

	UKBB background frequency (n with variant/n without)	UKBB frequency in individuals with UMOD c.278_289delTCTGCCCCGAAG insCCGCCTCCT variant (n with/n total)	P value	GEL background frequency (n with variant/n without)	GEL frequency in individuals with UMOD c.278_289delTCTGCCCCGAAG insCCGCCTCCT variant (n with/n total)	P value
16:19727140:AAAAG:A	329/115140	5/5	5×10^–12^	410/75211	16/20	<1×10^−16^
16:20420849:T:G	424/114716	4/5	1×10^–10^	228/75073	18/20	<1×10^−16^
16:20645695:T:G	418/114722	3/5	5×10^–7^	218/75083	18/20	<1×10^−16^
16:20794732:T:A	109/115031	4/5	4×10^–12^	76/75225	18/20	<1×10^−16^

Five of the nine individuals with c.278_289delTCTGCCCCGAAG insCCGCCTCCT variant in the UKBB had whole-genome sequencing data. Specific rare variants (<0.5% UKBB) in a 1 Mb window around the *UMOD* indel variant (column 1) were significantly over-represented in carriers of the *UMOD* variant versus the overall UKBB and 100K cohorts. The location of the variants are shown in figure 3. P values are based on Fisher’s exact test and only one individual from each family was included in the analysis.

GEL, Genomics England Limited; 100K, 100,000 Genomes Project; UKBB, UK Biobank; UMOD, uromodulin.

### Historical cases

Based on published papers, we identified a further 120 individuals with the indel variant drawn from 22 unique pedigrees (table 3). Of relevance, all of these cases originated from the UK and where stated, were of white British ethnicity.^8 35–38^

**Table 3:** Demographics of reported individuals with the c.278_289delTCTGCCCCGAAG insCCGCCTCCT *UMOD* variant in various cohorts

Study	Pedigrees (n)	Individuals (n)	Reference
Sheffield and Nottingham	12	88	This study
100K (GEL)	12	20	This study
UKBB	9	9	This study
Cambridge	5	35	37
Southampton	4	4	8
Cardiff (Cologne)	2	7	35 36
Wake Forest Registry, USA	11	74	7 19

GEL, Genomics England Limited; 100K, 100,000 Genomes Project; UKBB, UK Biobank; UMOD, uromodulin.

## Discussion

In this study, we provide compelling evidence that the *UMOD* variant c.278_289delTCTGCCCCGAAG insCCGCCTCCT, widely distributed within the UK population, is a founder variant. One hundred seventeen new individuals from 33 pedigrees were ascertained from clinical referrals within a limited geographic region of England, the 100K cohort and from the UKBB population. When published cases are included, a total of 237 individuals from 55 pedigrees have now been reported with this rare variant, all from the UK.

It is worth noting that the c.278_289delTCTGCCCCGAAG insCCGCCTCCT variant was the second most common change (14 pedigrees) reported in the International ADTKD patient registry of 722 individuals, and all cases were of white British ancestry^19^ (table 3). These observations are consistent with a founder effect. A previous single-centre study did not identify a common haplotype in four unrelated pedigrees based on limited analysis, the authors concluding that this was likely to be a recurrent change rather than a founder effect.^37^ Based on the number of cases since reported, the unusual nature of the variant, their restricted geographical and racial origin and now evidence of a shared haplotype, the evidence now points overwhelmingly to the existence of an ancestral mutation present in the UK population.

In the International ADTKD registry, there was no difference in the median age of kidney failure reported for patients with this variant (48 years) compared with the rest of the ADTKD-*UMOD* population (124 mutations; 47 years).^19^ The median age of kidney failure in our patients was however later (52 years) than previously reported. Although previous reports suggested longer kidney survival in females with ADTKD-*UMOD*,^19 39^ we did not detect a significant change in our clinical cohort. We also noted significant interfamilial and intrafamilial variability in the age of onset of kidney failure (range 32–76 years) implying that non-allelic and environmental factors can significantly modify kidney survival, as in other monogenic diseases.

Gout is a common feature in ADTKD-*UMOD*, being present in 50% of patients with a median age of onset of 28 years.^19^ The paucity of gout associated with this variant is striking when compared with other *UMOD* variants and has been noted previously.^35 37^ The absence of early onset gout as an alerting symptom is likely to have led to delayed clinical diagnosis but the absence of gout did not appear to impact overall kidney survival; hyperuricaemia is therefore unlikely to be a significant pathogenic factor for disease progression in ADTKD-*UMOD*. Hypertension was present in 65% of the clinical cases with available data and was also present in eight of the nine UKBB participants at baseline assessment (aged >48 years). Common *UMOD* variants have been associated with salt-sensitive hypertension, possibly by a functional interaction with the NKCC2 co-transporter.^40 41^ Only one patient had significant renal cystic disease (>10 cysts/kidney).

The indel sequence results in the deletion of five amino acids and a replacement with four residues (AlaAlaSerCys), disrupting the structure of the second calcium-binding EGF-like domain.^37^ The molecular mechanism underlying disease in ADTKD is more likely to relate to a dominant-negative or gain-of-function induced by the mutant protein rather than to loss-of-function.^9^ The intracellular accumulation of the mutant protein likely leads to ER stress and activation of the unfolded protein response pathway.^18 42^ Initial biochemical studies showed that the recombinant indel mutant protein showed a glycosylation pattern closer to the wild-type protein and was secreted more efficiently by transfected cells than another mutant (C150S).^37^ However, later assays by the same group using atomic force microscopy revealed that all three different *UMOD* variants tested (Indel, C150S, C155R) showed similar premature formation of intracellular fibrillar structures compared with the wild-type protein, despite the differences in glycosylation.^38^ These abnormalities are consistent with the similar age of kidney failure noted for this variant compared with others.^19^

## Conclusion

In summary, we report a pathogenic *UMOD* variant shared by 12 apparently unrelated families with familial kidney failure within a contiguous geographical region of England but which also appears to be distributed more widely within the UK. Our results demonstrate that this is likely to represent a common ancestral variant in unrelated families rather than a recurrent variant. The absence of early onset gout associated with this variant in comparison with classical *UMOD* variants likely led to a delay in diagnosis. Our paper highlights the need for genetic testing in all cases of familial CKD of uncertain aetiology. The *UMOD* variant c.278_289delTCTGCCCCGAAG insCCGCCTCCT should be suspected in all cases of familial nephropathy presenting in patients of white British ancestry.

## Data Availability

All data relevant to the study are included in the article or uploaded as supplementary information.
