# Supplementary material for: A founder UMOD variant is a common cause of hereditary nephropathy in the British population
Source: J Med Genet. 2022 Aug 29;60(4):397–405. doi: 10.1136/jmg-2022-108704 (PMC10086494; doi:10.1136/jmg-2022-108704)

Supplementary Table 1. Clinical features of patients with the c.278_289delinsCCGCCTCCT *UMOD* variant

	n (%)
CKD Staging	
No CKD	16 (18)
Median age (years)	30
CKD 3	7 (8)
CKD 4	4 (4.5)
Kidney failure	61 (69)
Median age (years)	52
Earliest onset of kidney failure (years)	32
Latest onset of kidney failure (years)	76
Renal Ultrasound	
Available imaging	28
Kidney length	
Normal (10-12cm)	17 (61)
Small (<10cm)	11 (39)
Cysts in each kidney	
No cyst	22 (79)
1 – 2	5 (18)
≥ 10	1 (4)
Hypertension	
Diagnosed	30 (65)
No hypertension	16 (35)
Unknown	42
Proteinuria	
A1 (<30mg/g)	36 (90)
A2 (30-300mg/g)	2 (10)
A3 (>300mg/g)	1 (5)
Not quantified	
Trace	1 (5)
Unknown	48
Haematuria	
No haematuria	33 (83)
Haematuria present	
Trace	3 (8)
1+	4 (10)
Unknown	48
Gout	
≥ 1 episode of gout diagnosed	6 (6.8)
Median age of onset (years)	42.5
No gout	82 (93)
Uric Acid	
Raised at presentation	9 (32)
Raised at least one instance	17 (61)
Never raised	11 (39)
Unknown	51
Renal stone	
Present	2 (4)
Absent	53 (96)
Unknown	33
UTI	
≥ 1 episode of symptomatic UTI	8 (15)
Absent	47 (85)
Unknown	33

Supplementary Table 2. Renal biopsy findings of patients with the c.278_289delinsCCGCCTCCT *UMOD* variant

Patient	Biopsy findings	Number of glomeruli affected	Immunofluorescence	Electron microscopy	Indication for biopsy
SN2.12	Glomerulonephritis and tubulointerstitial fibrosis	-	-	-	Unknown
SN3.1	Cellular interstitial aggregates of mixed origin. Significant renal scarring, interstitial fibrosis, tubular atrophy, marked vasculopathy. Tubulitis.<br><br>1 artery with significantly thickened wall and multiple elastic laminae layers. The arterioles show focal nodular hyalinosis.	5 of 11 glomeruli are obsolete.	No immune reactants.<br>-	No immune complex deposits. Only hyaline casts were present.	Proteinuria, CKD, Hypertension, Raised calcium (2.93)
SN3.2	Significant interstitial fibrosis and tubular atrophy.<br><br>Part of a medium sized artery is seen showing thickening of the wall.<br><br>Tamm-Horsfall protein casts seen without myeloma casts in tubules.	6 of 8 glomeruli globally sclerosed, remaining 2 showed peripheral fibrosis.	No immune reactants.<br>-		Proteinuria, CKD 3, Hypertension
SN3.5	Interstitial fibrosis and tubular atrophy.<br><br>One artery with marked chronic vasculopathy	3 of 12 glomeruli affected. 2 obsolete, 1 showed single podocyte with bubbly, clear, foamy cytoplasmic appearances.	-	Podocytes with increased numbers of enlarged lysosomes filled with myelinoid material.	Rapid renal function decline, raised IgG Kappa (5.5g/L) with slightly raised (6.7%) plasma cells on BM.
SN6.1	Excess glomerular loss and interstitial fibrosis. Glomerular hypertrophy<br><br>Normal arterial profile	7 of 14 globally sclerosed. Non sclerosed glomeruli are generally enlarged, no mesangial hypercellularity, segmental sclerosis	Minimal deposition of IgA within glomerular mesangium	No electron dense deposit or fibrillary material. Normal glomerular basement membrane. No	eGFR 46, no proteinuria, SOBOE, Hypertensive, Obese, Strong FHx of renal disease but related to diabetes in family members

				foot process effacement.	
SN8.5	Normal				
SN10.2	Advanced focal glomerulosclerosis and advanced tubulointerstitial fibrosis. Widespread tubular atrophy<br><br>Arterioles are thick walled but show no hyalinosis.	5 of 7 globally sclerosed. Non sclerosed glomeruli enlarged	No immune reactants.	No electron dense or fibrillary deposit. Mild wrinkling of capillary loops but normal glomerular basement membrane. Minimal foot process effacement.	Decline of renal function, FHx of renal disease, negative nephritic screen
SN11.1	Advanced glomerulosclerosis, interstitial fibrosis, heavy infiltrate of lymphocytes and plasma cells, tubular atrophy<br><br>Arteries are thick walled but without hyalinosis or intimal proliferation.	13 of 26 obsolete	No immune reactants.	Mild expansion of mesangial region, no proliferation Normal glomerular basement membrane. Minimal foot process effacement.	Strong family history of renal disease, declining renal function, biopsy 4 years ago showed non-specific chronic changes

Supplementary Table 3. Individuals in 100,000 Genome Project Cohort with the c.278_289delinsCCGCCTCCT *UMOD* variant

Family ID	Proband	Sex	Renal	Disease	Phenotypes
GE1	Yes	Female	Yes	CAKUT	CKD (HP:0012622), Hypertension (HP:0000822), Obesity (HP:0001513), Polycystic ovaries (HP:0000147), Gluten intolerance (HP:0012538)
GE1	No	Female	Yes	CAKUT	CKD (HP:0012622), Hyperparathyroidism (HP:0000843)
GE2	Yes	Male	Yes	Proteinuric renal disease	CKD (HP:0012622), Hypertension (HP:0000822)
GE2	No	Male	Yes	Proteinuric renal disease	
GE3	Yes	Female	Yes	Proteinuric renal disease	Polycystic kidney dysplasia (HP:0000113), Proteinuria (HP:0000093), Obesity (HP:0001513)
GE3	No	Female	Yes	Proteinuric renal disease	Polycystic kidney dysplasia (HP:0000113), Proteinuria (HP:0000093), Diabetes Mellitus (HP:0000819)
GE4	Yes	Male	No	Intellectual disability	Autistic behaviour (HP:0000729), Global developmental delay (HP:0001263)
GE4	No	Male	No		
GE5	Yes	Male	No	Intellectual disability	Delayed speech and language development (HP:0000750), Global developmental delay (HP:0001263)
GE5	No	Female	No	Intellectual disability	Delayed speech and language development (HP:0000750), Inability to walk (HP:0002540)
GE5	No	Male	No		
GE6	Yes	Female	Yes	Unexplained kidney failure	Nephrosclerosis (HP:0009741), Proteinuria (HP:0000093), Edema (HP:0000969)
GE6	No	Male	Yes	Unexplained kidney failure	Stage 4 CKD (HP:0012626), CKD (HP:0012622), Hypertension (HP:0000822), Multiple renal cysts (HP:0005562)
GE7	Yes	Female	Yes	Familial haematuria	Stage 4 CKD (HP:0012626), Renal cortical atrophy (HP:0002048), Microscopic hematuria (HP:0002907), Gout (HP:0001997)
GE7	No	Female	Yes		Stage 3 CKD (HP:0012625), Hematuria (HP:0000790)
GE8	Yes	Male	No	Hypertrophic Cardiomyopathy	Hypertension (HP:0000822), Dyspnea (HP:0002094), Hypertrophic cardiomyopathy (HP:0001639)
GE9	Yes	Male	Yes	Unexplained kidney failure	CKD (HP:0012622), Renal atrophy (HP:0012585)
GE10	Yes	Female	No	Adult Glioma	
GE11	Yes	Male	Yes	Renal Cancer	
GE12	Yes	Male	No	Prostate Adenocarcinoma	

Figure S1

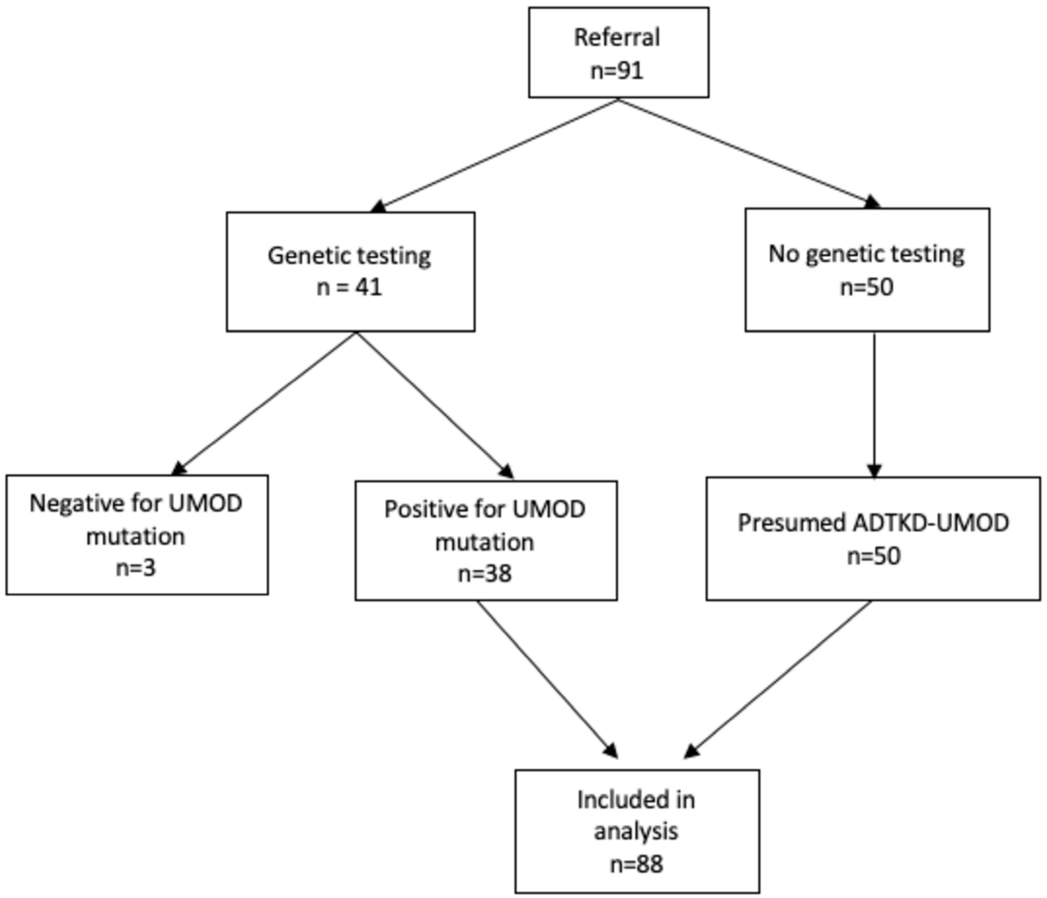
Referral
n=91
Genetic testing
n = 41
No genetic testing
n=50
Negative for UMOD
mutation
n=3
Positive for UMOD
mutation
n=38
Presumed ADTKD-UMOD
n=50
Included in
analysis
n=88

BMJ Publishing Group Limited (BMJ) disclaims all liability and responsibility arising from any reliance placed on this supplemental material which has been supplied by the author(s)

Figure S2

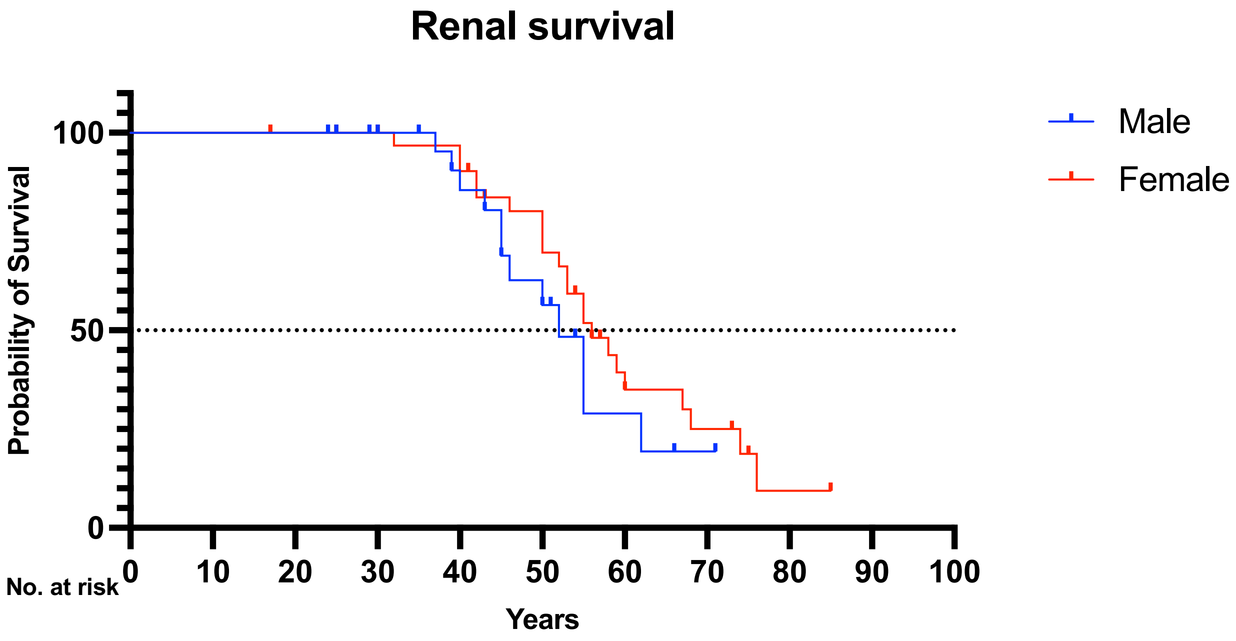

**Male**	27	27	27	24	18	10	3	1	N/A
**Female**	35	35	35	32	30	23	9	5	1

Figure S3

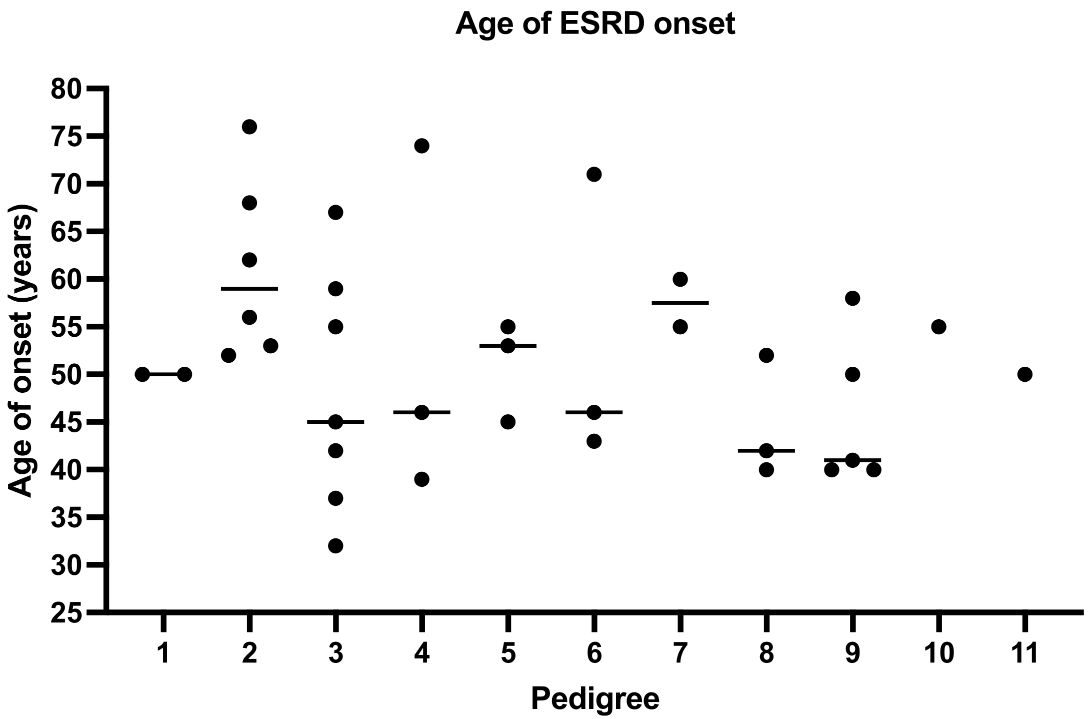
Age of ESRD onset
Age of onset (years)
80
75
70
65
60
55
50
45
40
35
30
25
1
2
3
4
5
6
7
8
9
10
11
Pedigree

Figure S4

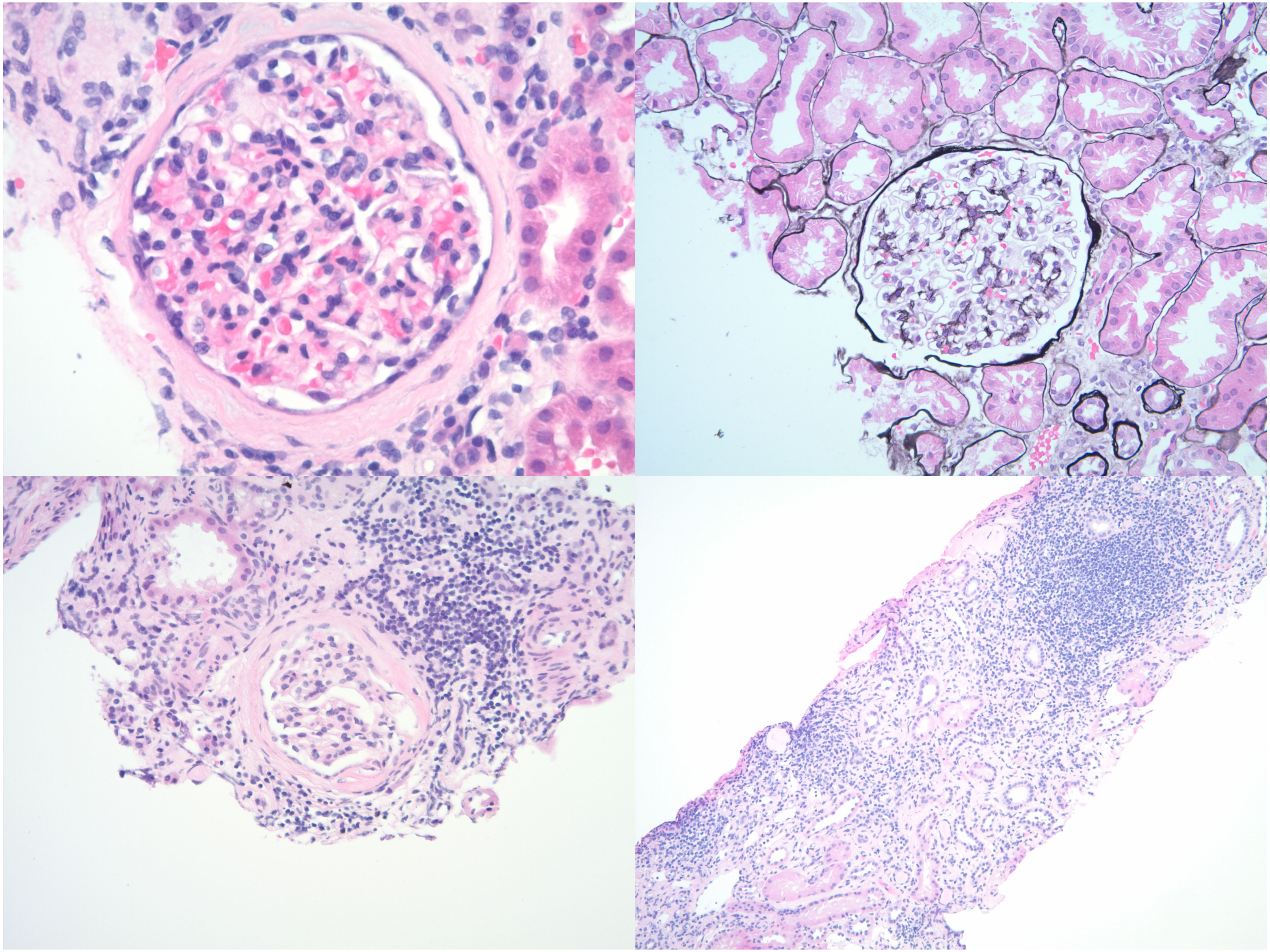

3.1

3.2

Sheffield Cohort

Figure S5

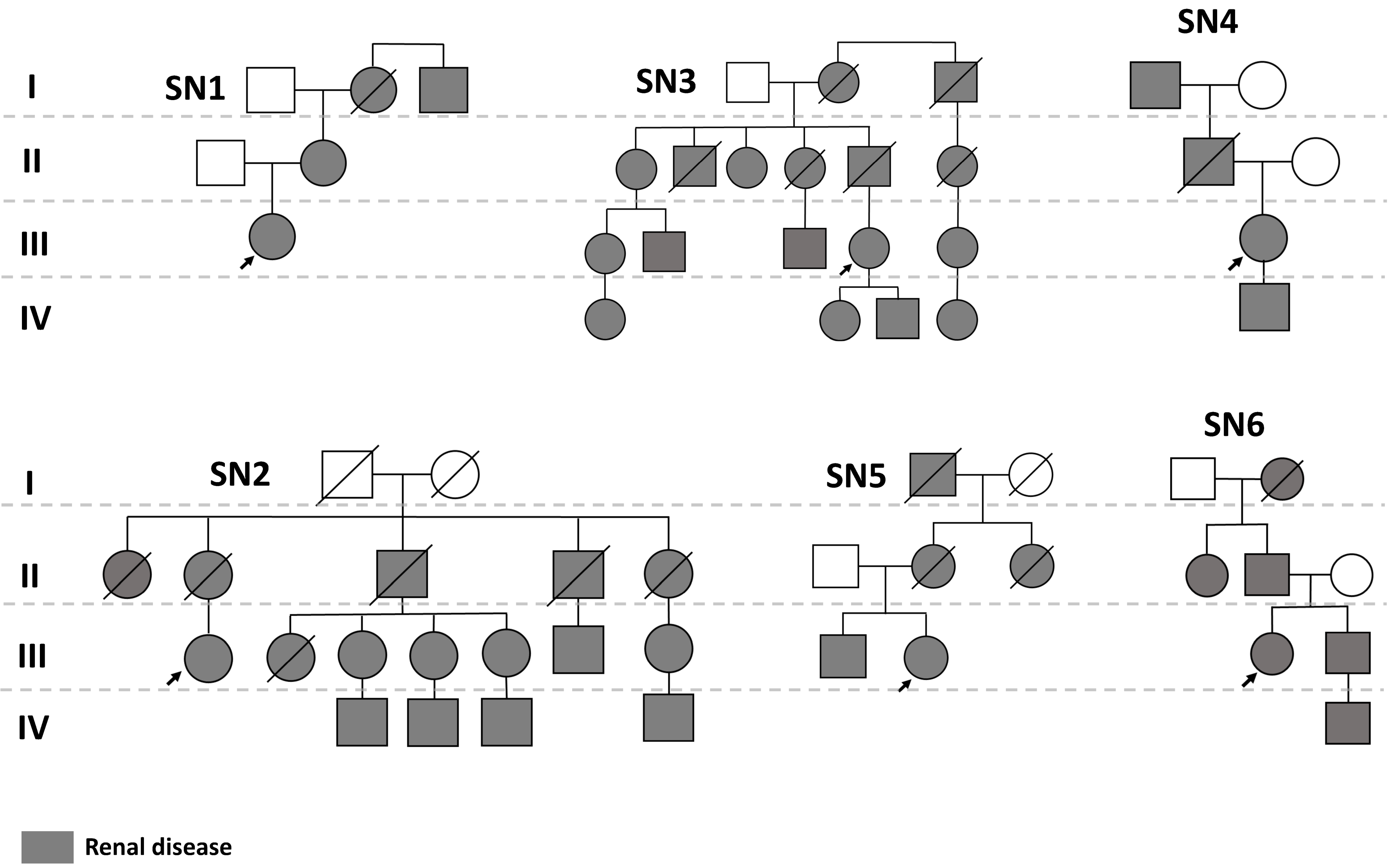

Nottingham Cohort

Figure S6

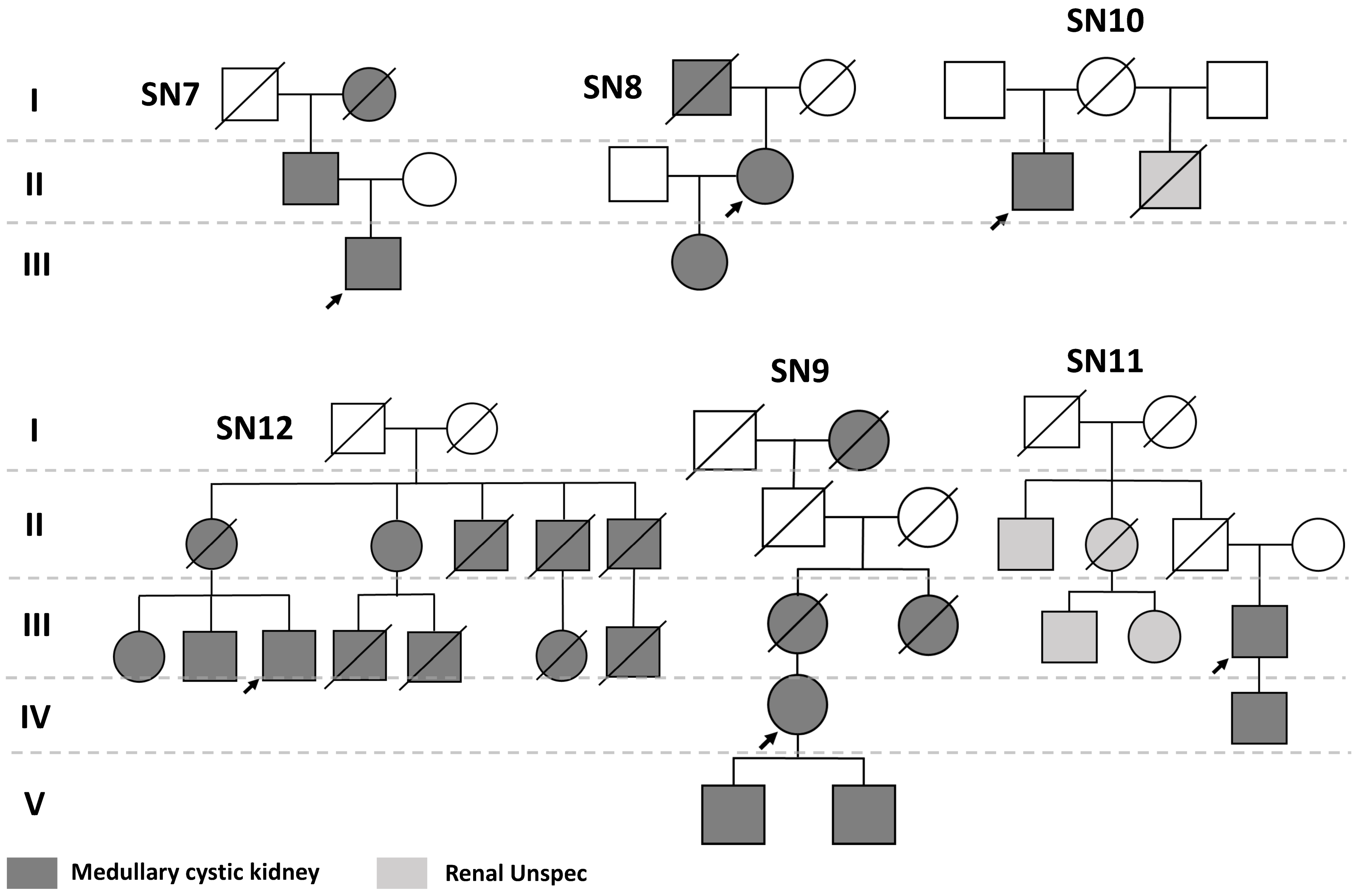

Figure S7

100k Genomes Project

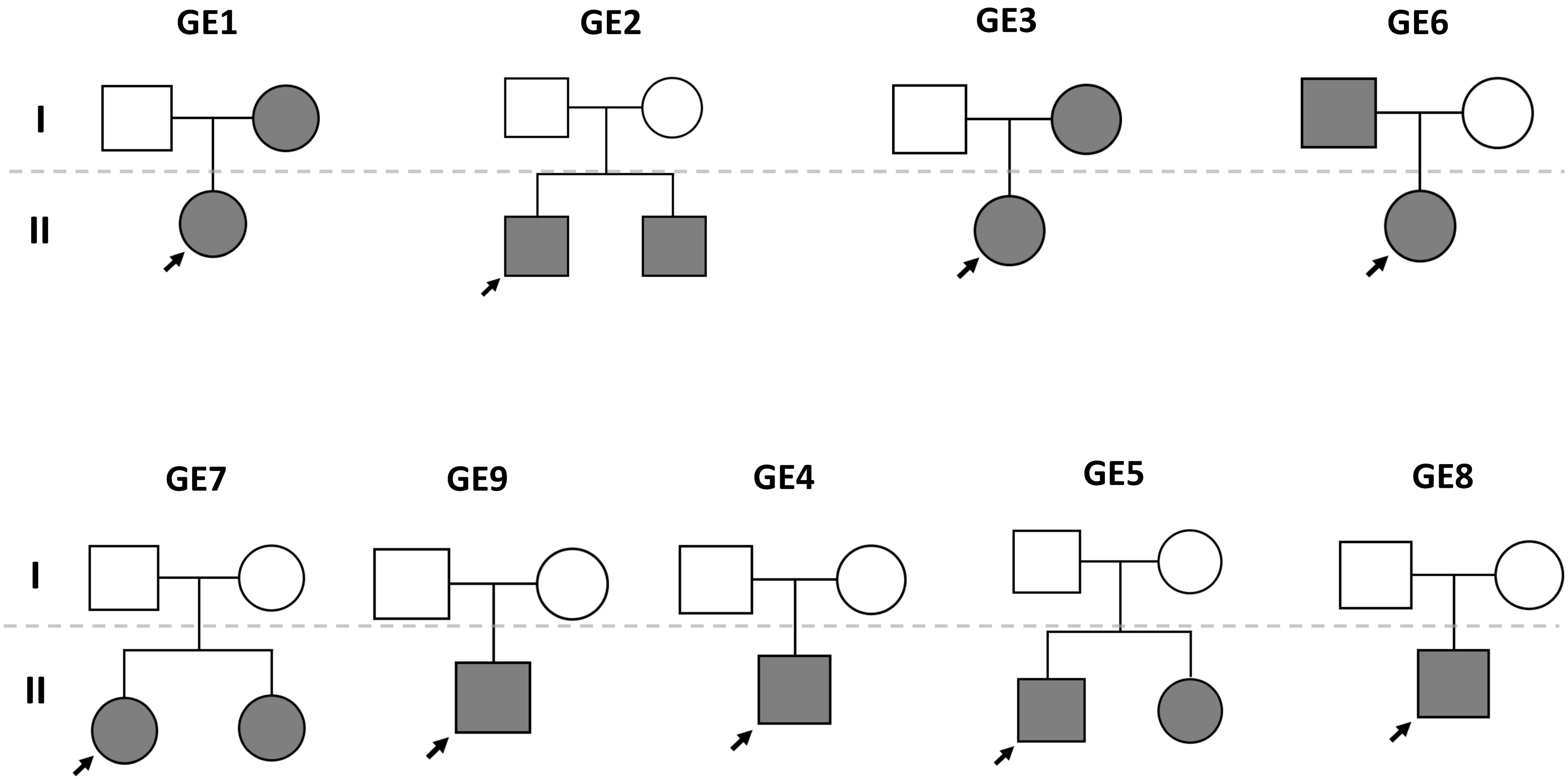

Supplement: Supplementary data [file jmg-2022-108704supp001.pdf]
